# Xiaoyin-anshen formula alleviates psoriasis complicated by sleep disturbances by regulating melatonin, antioxidant enzymes, and pro-inflammatory cytokines in mice

**DOI:** 10.3389/fphar.2024.1427985

**Published:** 2024-10-01

**Authors:** Zebing Zhu, Qiang Yin, Xingwu Duan

**Affiliations:** ^1^ Department of Dermatology, Dongzhimen Hospital, Beijing University of Chinese Medicine, Beijing, China; ^2^ Hubei Provincial Hospital of Traditional Chinese Medicine, Wuhan, Hubei, China

**Keywords:** melatonin, oxidative stress, psoriasis, pro-inflammatory cytokines, RORα, sleep disturbances, Traditional Chinese Medicine

## Abstract

**Background:**

Psoriasis is a common autoimmune and chronic inflammatory dermatological disease that is mainly associated with aberrant immune response and oxidative stress (OS). OS, a crucial pathogenic factor in psoriasis, contributes to psoriasis-like inflammation mediated by the IL-23/IL-17 inflammatory axis. Sleep disturbances (SDs), highly prevalent in patients with psoriasis, exacerbate the condition by disrupting circadian rhythms and reducing melatonin levels, thus promoting OS and inflammation. Xiaoyin-Anshen formula (XYAS), a traditional Chinese medicine (TCM) formula, is composed of the Liangxue-Jiedu (LXJD) and Qingxin-Anshen (QXAS) TCM compounds and has been demonstrated to be effective in treating psoriasis complicated by SDs. However, its exact pharmacological mechanism remains uncertain. Thus, this study used animal experiments to verify whether XYAS can exert therapeutic effects on the disease by regulating melatonin (MLT) levels, protecting against OS, and inhibiting psoriasis-like skin inflammation.

**Methods:**

A mouse model for psoriasis combined with SDs was established by smearing 62.5 mg of 5% imiquimod (IMQ) cream for seven consecutive days, along with a daily injection of p-chlorophenyl alanine (PCPA) solution at a dosage of 300 mg/kg at days 6–7. The IMQ cream was continued to be used for maintaining the model at days 8–14. Mice were randomly divided into groups: control, model, MLT, XYAS, LXJD, QXAS. Each group was treated according to its designation at days 8–14, receiving either an oral gavage of XYAS/LXJD/QXAS solution at a dosage of 2 mL/100 g per day, or a daily injection of MLT solution at a concentration of 0.25 mg/mL, with a dosage of 5 mg/kg. Immunohistological analysis, pentobarbital-induced sleep test, Western blotting, and enzyme-linked immunosorbent assay (ELISA) were performed to assess and compare pathological features, sleep conditions, localization and/or levels of manganese-dependent superoxide dismutase (mnSOD), mitochondrial cytochrome c (Cyt-C), MLT, retinoid-related orphan nuclear receptor-α (RORα), and pro-inflammatory cytokines interleukin (IL)-6, IL-17A, and tumor necrosis factor-alpha (TNF-α) among groups.

**Results:**

MLT, XYAS, LXJD, and QXAS exhibited varying therapeutic effects on RORα regulation, OS inhibition, mitochondrial protection, and anti-inflammation. Compared to the model, the lesion severity/thickness and serum IL-6, IL-17A, and TNF-α levels were gradually reduced in the MLT, QXAS, LXJD, and XYAS. However, no statistical difference in TNF-α levels was identified between the MLT and the model groups. Additionally, skin MLT levels gradually increased in the MLT, QXAS, and XYAS groups, while RORα levels gradually increased in the MLT, QXAS, LXJD, and XYAS groups. All treatments increased mnSOD levels and reduced Cyt-C levels in skin lesions, with XYAS showing the most significant changes.

**Conclusion:**

XYAS may treat psoriasis complicated by SDs through two main mechanisms: (1) Improving melatonin-RORα axis in the skin can lead to an increase in mnSOD and a reduction in Cyt-C levels, which provide protection against oxidative stress, mitochondrial damage, and psoriatic inflammation. (2) Reducing IL-6, IL-17A, and TNF-α production to suppress IL-23/Th17 pro-inflammatory signaling axis and epidermal hyperplasia in psoriasis.

## 1 Introduction

Psoriasis is an autoimmune and chronic inflammatory dermatological disease that frequently occurs in approximately 76% of psoriasis patients suffering from sleep disturbances (SDs) (Henry et al., 2017). Psoriasis is believed to cause SDs and pruritus due to its characteristics of cytokines and neuropeptides dysregulations, which activate the immunological-inflammatory cascades (Luca et al., 2016); (Lavery et al., 2016). And both psoriasis and SD can exacerbate each other, forming a bidirectional pathogenic loop (Woo et al., 2020). Specifically, oxidative stress (OS) can stimulate the secretion of pro-inflammatory cytokines such as tumor necrosis factor (TNF)-α, interferon-gamma (IFN-γ), interleukin (IL)-17, IL-22, and IL-23. It also induces Th1 and Th17 differentiation and decreases regulatory T cells (Tregs) activity, thereby playing a crucial role in psoriasis- and SD-associated inflammation (Hill et al., 2018; Wu et al., 2020; Lai et al., 2018; Zhou et al., 2016). Conversely, psoriasis can exacerbate OS by promoting pro-oxidative processes and pro-inflammatory cytokines generation (Wójcik et al., 2019). SDs, often accompanied by decreased melatonin (MLT) secretion and disrupted circadian rhythm (Vasey et al., 2021), further aggravate OS and upregulate the expression of pro-inflammatory cytokines such as IL-6 and TNF-α (Mullington et al., 2009; Hirotsu et al., 2012; Prather et al., 2009). Current treatment strategies in modern medicine emphasize etiological treatment to alleviate SDs and psoriasis, such as benzodiazepines for sleep and biologics targeting IL-17, IL23, or TNF-α that inhibit IL-23/Th17 pro-inflammatory signaling axis and improve antioxidant capability to suppress chronic inflammation and halt the progression of psoriasis (Mihu et al., 2021; Blagov et al., 2023). Despite these advancements, several significant limitations persist, including safety concerns, side effects, resistance to treatment, single target, and recurring inflammation (Lee and Kim, 2023; Lavigne et al., 2019). Thus, there is an urgent need for an efficient, safe, and cost-effective clinical therapy for this disease.

Traditional Chinese medicine (TCM) formulae are known for their ability to target multiple sites, high efficacy, few side effects, and accurate syndrome differentiation in symptomatic treatment. We observed that Xiaoyin-Anshen formula (XYAS) had remarkable clinical efficacy against the co-occurrence of psoriasis and SDs, but its pathological mechanism remains unclear. The development of XYAS was based on Xiaoyin-Jiedu decoction (XYJD). Specifically, Baixianpi (Cortex Dictamni Radicis) and Tufuling (Rhizoma Smilacis Chinae) were deleted and Qingxin-Anshen prescription (QXAS) were added to XYJD to produce XYAS, which was utilized in the treatment of psoriasis complicated by SDs. The QXAS was for cooling and detoxifying the blood, calming the mind, and clearing the heart. We confirmed the therapeutic effect of XYJD on psoriasis, demonstrating its ability to significantly decrease the protein and mRNA expression levels of Th-17-related IL-23, IL-22, IL-17, and retinoid-related orphan nuclear receptor (ROR)-γt while balancing the Th17/Tregs in the peripheral blood of patients with psoriasis patients (Chen et al., 2020). Additionally, Ziziphi Spinosae Semen, Radix Salviae, Polygala tenuifolia Willd and Coptidis Rhizoma in QXAS have been proven to improve insomnia (Yang et al., 2023; Li et al., 2022; Zhang D. et al., 2023; Sun et al., 2023). Notably, Coptidis Rhizoma treatment can increase the secretion of MLT and reduce the production of reactive oxygen species (Xia et al., 2022; Kwon et al., 2016). Polygala tenuifolia Willd and Radix Salviae are rich in MLT (Chen et al., 2003), while Ziziphi Spinosae Semen regulates circadian rhythm, increases MLT synthesis, and protects against OS and inflammatory response (Zhang et al., 2024). Although Park et al., reported that the expression of imiquimod (IMQ)-activated nuclear factor kappa-B (NF-κB) was decreased in RORα−/− mice compared to RORα^+/+^ mice (Park et al., 2023). The role of RORα in the pathogenesis of psoriasis has not been fully elucidated yet. Moreover, both MLT and RORα can modulate sleep and circadian rhythm, and it has been proven that MLT relies on RORα to improve mitochondrial dysfunction (Wang et al., 2023), mitigate NF-κB-induced inflammatory response, and offer protection against OS (Ma et al., 2021; García et al., 2015). These findings suggest that the MLT-RORα axis may represent a promising therapeutic target for psoriasis.

Considering the therapeutic effects of XYAS and the bidirectional interaction between psoriasis and SDs (Nowowiejska et al., 2021), it is crucial to explore its specific pharmacological mechanism involving the regulation of the MLT-RORα axis, pro-inflammatory cytokines, and OS in psoriasis. Through animal experiments, we discovered that XYAS could upregulate the levels of MLT-RORα and antioxidant enzyme in skin while decreasing mitochondrial damage and inflammation in psoriasis combined with SDs. This study contributes to a better understanding of whether and how XYAS can alleviate psoriatic inflammation resulting from the vicious cycle of psoriasis and SDs, thereby providing an experimental foundation for the clinical application of XYAS.

## 2 Materials and methods

### 2.1 Experimental animals and design

Sixty SPF male Balb/C mice (SCXX Beijing 2015–0001), weighing 18–20g, were obtained from Weitong Lihua Laboratory Animal Technology Co., Ltd. (Beijing, China), and housed in a pathogen-free animal room (SPF grade) at Dongzhimen Hospital. The experimental protocol was reviewed and approved by the Animal Research Ethical Committee at Dongzhimen Hospital of Beijing University of Chinese Medicine. After 1 week of adaptive feeding, the mice were dehaired to expose a back area of approximately 2 × 3 cm and then randomly divided into six groups, each consisting of 10 mice. Group 1 was served as the healthy control, receiving medical Vaseline on the exposed area (once daily, at days 1–14), intraperitoneal injections of normal saline (2 mL/100g, once daily, days 6–7), and an oral gavage of physiological saline (once daily, 2 mL/100g, at days 8–14). Group 2 was the model group and groups 3–6 were served as treatment groups. These groups were smearing with 65 mg of 5% IMQ cream on the back to induce and maintain psoriasis-like inflammation (once daily, at days 1–7 for inducing and days 8–14 for maintaining psoriasis) (Liang et al., 2020)and intraperitoneally injected with p-chlorophenyl alanine (PCPA) solution to lead SDs (once daily, 300 mg/kg, days 6–7) (JinLv et al., 2021; Satinoff et al., 1991). After 1-week continuous usage of IMQ cream, mice in group 2 were given physiological saline (once daily, 2 mL/100g, at days 8–14), mice in groups 3, 4, 5 were orally gavaged with 0.5 g/mL of XYAS or LXJD or QXAS solutions at a dosage of 2 mL/100 g (calculated from human dosage, once daily, at days 8–14) (Jin et al., 2021), mice in group 6 were intraperitoneally injected with MLT solution at a concentration of 0.25 mg/mL (once daily, 5 mg/kg, at days 8–14) (Czarnecka et al., 1999). On day 15, the eyeballs of mice were removed to collect blood samples.Then, the mice were sacrificed via cervical dislocation and their back-skin tissues were harvested for later use.

### 2.2 Composition and preparation of XYAS formula

The XYAS was developed by Professor Xingwu Duan for the target management of psoriasis complicated by SDs. The XYAS formula is composed by two components: LXJD and QXAS formulae. The compositions were described as follow. LXJD: Buffalo Horn Extract (Shuiniujiao in Chinese, Horn Extract of *Bubalus bubalis Linnaens*, No. T000202395, Beijing, China; 30 g), Rehmanniae Radix (Shengdihuang in Chinese, Dried Root of *Rehmannia glutinosa Libosch*, No. T110202800, Beijing, China; 20 g), Paeoniae Radix Rubra (Chishao in Chinese, Dried Root of *Paeonia lactiflora Pall*, No. T330201992, Zhejiang, China; 12 g), Cortex Moutan (Mudanpi in Chinese, Dried Root bark of *Paeonia suffruticosa Andr*, No. T000200576, China; 12 g), *Lonicera Japonica* Caulis (Rendongteng in Chinese, Dried Sterm of *Lonicera japonica Thunb*, No. T330203510, Zhejiang, China; 10 g), Isatidis Folium (Daqingye in Chinese, Dried Leaves of *Isatis indigotica Fort*, No. T000200183, China; 15 g). QXAS: Polygalae Radix (Yuanzhi in Chinese, Dried Root of *Polygala Tenuifolia Willd*, No. T001400857, China; 10 g), Salviae Miltiorrhizae Radix Et Rhizoma (Danshen in Chinese, Dried Root and Rhizome of *Salvia miltiorrhiza Bge,* No. T001200177, China; 15 g), Coptidis Rhizoma (Huanglian in Chinese, Dried Rhizome of Coptis chinensis Franch, No. T000200367, China; 10 g), Glycyrrhizae Radix Et Rhizoma (Shenggancao in Chinese, Dried Root and Rhizome of *Glycyrrhiza uralensis Fisch*, No. T001700276, China; 10 g), Ziziphi Spinosae Semen (Suanzaoren in Chinese, Dried mature seeds of Ziziphus jujuba Mill. Var, No. T001400719, China; 10 g), Ganoderma powder (Lingzhi in Chinese, Dried Fruiting bodies of *Ganoderma lucidum* (*Leyss.ex Fr.*) *Karst*, No. T111701154, Beijing, China; 10 g). The Chinese herbal medicines were identified and supplied by the TCM pharmacy of Dongzhimen Hospital, Beijing University of Chinese Medicine. The medicines were prepared using boiling extraction (decocting), which is the most common and earliest method for TCM preparation (Gao et al., 2021). The decoctions were prepared in accordance with the Standard for Management of TCM Decocting Room in Medical Institutions. Specifically, herbal medicines were soaked in 5 times their volume of water for 0.5 h before boiling for 2 h. Subsequently, an equal volume of water was added, and the mixture was boiled for another 0.5 h. The mixture was then filtered, and the filtrate was sterilized. A final concentration of 0.5 g/mL was obtained and the mixture was stored at 4°C.

### 2.3 Preparation of experimental reagents

IMQ cream (CAS. No. 99011–02–6; Batch number: National Drug Approval Letter H20030128) was purchased from Mingxin Pharmaceutical Co., Ltd (Sichuan, China). PCPA (CAS. No. 1878–66–6) was purchased from Sigma-Aldrich (St.Louis, United States). Medical Vaseline was from Baiyun Pharmaceutical Co., Ltd (Nanchang, China). Pentobarbital sodium salt (CAS. No. 57–33–0) and Melatonin (CAS. No. 73–31–4) were purchased from Sigma-Aldrich (St.Louis, United States). Hematoxylinandeosin (HE) staining (D006) was purchased from Nanjing Jiancheng Bioengineering Institute (Nanjing, China). The primary antibodies for immunohistochemical staining included anti-RORα antibody (ab256799, Abcam, UK), anti-mnSOD antibody (ab13534, Abcam, UK), anti-Cyt-C antibody (ab133504, Abcam, UK). And the secondary antibodies were goat anti-mouse IgG polyclonal antibody (C1308, Pulilai Gene Technology Co., Beijing, China) and goat anti-rabbit IgG polyclonal antibody (C1309, Pulilai Gene Technology Co., Beijing, China). The primary antibodies used for Western blot were anti-RORα (ab256799, Abcam, UK), anti-cytochrome c (Cyt-C) (ab133504, Abcam, UK), anti-mnSOD (ab13533, Abcam, UK), and the anti-beta actin Monoclonal antibody (66009-1-IG, Proteintech, China) was used as internal reference. The bicinchoninic acid (BCA) protein determination reagent (Lot number: P1511) and RIPA lysis buffer (Lot number: C1053) were purchased from Beijing PuLilai Gene Technology Co., Ltd., Beijing, China. The reagents used for ELISA include mouse Melatonin ELISA kit (ab285251, Abcam, UK),mouse IL-6 ELISA kit (KE100007, Proteintech, United States), mouse IL-17a ELISA kit (KE10020, Proteintech, United States), and mouse TNF alpha ELISA kit (KE10002, Proteintech, United States). Other chemicals, included 5X protein loading buffer (B1030), Tris-Hcl Ph7.4 (B1010) and antibody diluent (C1240), were provided by Preley Gene Technology Co., Ltd (Beijing, China).

### 2.4 Scoring for back-skin lesions

Dorsal lesions were assessed based on the psoriasis area and severity index (PASI) scoring standard. The human PASI standard was used as a reference for scoring the severity of psoriasis in the mice due to a lack of specific scoring criteria for mice. Specifically, psoriasis was evaluated using the sum of the intensity scores for scaling, erythema, infiltration, and thickness. For each symptom, the score ranged from 0 to 4, with higher scores indicating more severe disease (Zhang et al., 2021). The difference in mean PASI score between the groups was analyzed to assess the efficacy of XYAS in psoriasis.

### 2.5 Pentobarbital sodium-induced sleep test

The severity of SDs in the mouse model was evaluated using the pentobarbital sodium-induced sleep test, following the methods outlined by (Yang et al., 2017). The test was carried out approximately 18–30 h after the first injection of PCPA. The mice were intraperitoneally injected with sodium pentobarbital (dissolved in saline at 10 mg/mL) at a dose of 35 mg/kg. Sleep latency and sleep duration were examined in this test. Sleep latency was defined as the period from the injection of pentobarbital to the loss of righting reflexes, and sleep duration was considered as the time interval between the recovery and loss of righting reflexes.

### 2.6 Histopathological and immunohistochemical assays for dorsal skin lesions

The histopathological features and the immunohistochemical localization of RORα, manganese-dependent superoxide dismutase (mnSOD), and Cyt-C in lesion tissues were examined. Briefly, 4% paraformaldehyde-fixed and paraffin-embedded back-skin tissue sections were stained with HE staining solutions, and photographs were recorded using the Olympus CH-20 optical microscope (Tokyo, Japan) and analyzed using the ImageJ software. Additionally, the skin sections were also subjected to immunohistochemical assays. The sections were then hybridized with the pre-diluted primary antibodies (anti-RORα, anti-mnSOD, or anti-Cyt-C) after being deparaffinized, hydrated, and washed with phosphate-buffered saline. Subsequently, the sections were treated with the corresponding secondary antibodies, either Goat Anti-Mouse IgG or Goat Anti-Rabbit IgG, as per the instructions provided with the primary antibodies. Sections from each group were then visualized and images were captured using an Olympus CH-20 optical microscope at a magnification of ×100. These images were subsequently analyzed using ImageJ. Positive results for RORα, mnSOD, and Cyt-C expressions in tissues were indicated by the presences of rich brown-yellow pigments staining the nuclei of the cells.

### 2.7 Enzyme-linked immunosorbent assay

The concentrations of MLT in lesion tissues and IL-6, IL-17a, and TNF-α in serum were determined using specific ELISA kits for mice. The mouse melatonin ELISA kit (ab285251, Abcam) was used for MLT detection, while the mouse IL-6 ELISA kit, mouse IL-17a ELISA kit, and mouse TNF alpha ELISA kit were used for IL-6, IL-17a, and TNF-α detection, respectively. The assay was conducted according to protocol provided by the manufacturer, and the target protein concentrations were calculated based on the optical density (OD) values of the proteins measured at 450 nm using a microplate reader.

### 2.8 Western blotting

Approximately 50 mg of each lesion tissue was collected and cut into pieces on ice. Protein lysates were prepared from these tissues by mixing with RIPA buffer supplemented with phosphatase and protease inhibitors. The concentration of the protein lysate supernatant was quantified using a bicinchoninic acid (BCA) protein assay kit. Equal amounts of protein from each sample were separated using sodium dodecyl sulfate-polyacrylamide gel electrophoresis (SDS-PAGE) and transferred onto a polyvinylidene fluoride (PVDF) membrane. The membrane was subsequently blocked with 5% skimmed milk. After blocking, the PVDF membranes were incubated with diluted primary antibodies (anti-RORα, anti-Cyt-C, and anti-mnSOD) overnight at 4°C. ß-actin was served as a loading control. After washing, the PVDF membranes were incubated with the corresponding secondary polyclonal antibodies (Goat Anti-Mouse IgG or Goat Anti-Rabbit IgG) for 1 h at room temperature. An ultra-high sensitivity ECL Kit (1060, LABLEADE) was used to visualize the antigen-antibody reaction, and the band signals of target proteins were examined by a Gel imager. The expression levels of RORα, Cyt-C, and mnSOD were calculated as the target protein gray value/internal reference using ImageJ.

### 2.9 Statistical analysis

Data were analyzed using SPSS 26.0 (SPSS Inc., Chicago, IL, United States) and GraphPad Prism (version 9), and presented as the mean ± standard deviation or median (interquartile range). The independent sample *t*-test was applied for comparisons between two groups of data with normal distribution, whereas Mann-Whitney U-test was applied when the data did not follow normal distribution. Additionally, one-way ANOVA was used for comparisons between multiple groups in cases where the variance was homogeneous, whereas the Kruskal–Wallis rank-sum test was used in cases where the variance was not homogeneous. The threshold for significance was set at *P*< 0.05.

## 3 Results

### 3.1 Characteristics of lesions and sleep performance in a mouse model of psoriasis complicated by SDs

**FIGURE 1:**
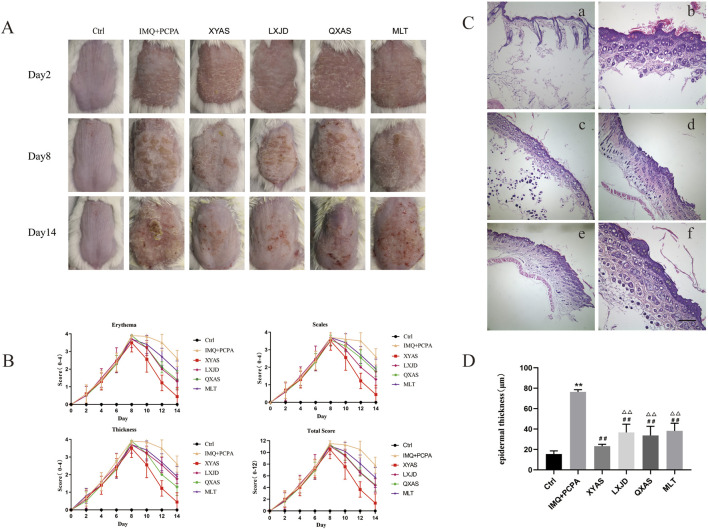
XYAS, LXJD, QXAS, and MLT improved psoriasis-like skin inflammation in lesion tissues of mice treated with IMQ cream and PCPA solution **(A)** Representative dorsal skin images of each group shoot on days 2, 8, and 14. **(B)** Variations in total PASI scores and scores for erythema, scaling, and thickness of each group throughout the entire experiment. **(C)** Representative HE staining images of skin tissues in each group (magnification×100): In **(C)**, a is the Control group (Ctrl), b is the IMQ + PCPA group, c is the XYAS group, d is the LXJD group, e is the QXAS group, f is the MLT group. **(D)** Epidermal thickness of lesion tissues in each group: ^**^ *p* < 0.05 or ^**^ *p* < 0.01, comparisons with the control group; ^##^ *p* < 0.01, comparison with the IMQ + PCPA group; ^△△^ *p* < 0.01, comparison with the XYAS group.

IMQ cream was applied onto the exposed dorsal skin for 14 days, and PCPA solution was intraperitoneally injected at days 6–7 to establish a mouse model for psoriasis combined with SDs. As depicted in Figures 1A, B, the control group exhibited normal skin conditions throughout the experiment, characterized by the absence of erythemas, scales, and abnormal thickness. In contrast, the IMQ + PCPA group (model) showed initial signs of light red spots with a slight amount of scales at day 2. These psoriatic lesions began to significantly worsen at day 4. Since then, the lesions gradually thickened, initially presenting as light pink spots, and progressed to extensive areas of moderate-to-severe erythema. Additionally, the mild scaling evolved into lamellar scales that covered the exposed skin. After receiving 2 days of PCPA injection, the IMQ + PCPA group presented with the most severe psoriatic symptoms and its PASI score reached the peak at day 8. From day 9, there was a gradual reduction in scales, thickness, and erythema, albeit some lesions remained reddish, slightly above normal skin surface and covered with moderate scales until the end point of the experiment.

Additionally, 28–30 h after the first PCPA injection, a pentobarbital sodium-induced sleep test was conducted. We observed that the model group exhibited significantly longer sleep latency and reduced sleep duration compared to the control group (Table 1). The mice in control group maintained normal circadian rhythms, and their hair stayed smooth and shiny (Figures 1A, B). However, mice in the IMQ + PCPA group exhibited rough, messy, dull hair and abnormal circadian rhythms. These mice also exhibited increased excitability, daytime activities, and sensitivity to external stimulation. Taken together with the observed skin features and PASI scores, these findings suggest that the mouse model established through the combined application of IMQ cream and PCPA solution effectively replicates defining characteristics of psoriasis and SDs, with the latter exacerbating the IMQ-induced psoriasis-like inflammation.

**TABLE 1:** The comparison of sleep performance between groups control and model in the pentobarbital-induced sleep tes**t** (mean ± standard deviation) or (median, interquartile range).

Groups	Sleep latency (min)	Sleep duration (min)
Control	42.50 ± 2.68	45.14 ± 2.12
IMQ + PCPA	48.6 (7.20)	35.13 ± 1.48
*Z/t*	−2.887	9.483
*P*	0.002	0.000

### 3.2 MLT, XYAS, LXJD, and QXAS can improve the macroscopic and histopathological appearances of psoriatic lesions in mice treated with IMQ + PCPA

The IMQ + PCPA-induced mouse model was used to examine the therapeutic effects of MLT, XYAS, LXJD, and QXAS on psoriatic lesions complicated by SDs. As depicted in Figures 1A–D, the model and treatment groups initially presented severe erythematous plaques covered with layers of scales on markedly thickened and infiltrated skin, leading to peak PASI scores at day 8. Since day 8, the XYAS, LXJD, QXAS, and MLT groups received the corresponding treatments until the conclusion of the experiment. Throughout the treatment period, the severity of psoriatic skin lesions and PASI scores in these groups were gradually reduced. Specifically, mice in the XYAS group exhibited significantly improved psoriatic symptoms compared to those in the LXJD, QXAS, and MLT groups. At day14, we observed that mice in the XYAS group exhibited mild erythemas, slight scales, and relatively smooth skin surfaces with no apparent thickening of the epidermis. The mice in XYAS group also demonstrated a significantly decreased PASI score compared to those in the LXJD, QXAS, and MLT groups. Notably, after the entire treatment duration, mice in the LXJD group and QXAS groups exhibited almost same level improvement in psoriatic lesions and erythema, with nearly identical PASI scores and erythema scores. However, the LXJD group exhibited a notable reduction in scales, with a lower scale score, and a less remarkable improvement in epidermal thickness, with a higher thickness score compared to the QXAS group. The MLT group also exhibited improvements in erythema, scales, and epidermal thickness. But these improvements were not as significant as those observed in the XYAS, LXJD, and QXAS groups. Consequently, the MLT group had higher PASI scores compared to the QXAS, LXJD, and XYAS groups.

Moreover, we conducted a histopathological examination of the mice using HE staining (Figure 1C). On day 14, the cell morphology of back-skin tissue remained normal in the control group. In contrast, the lesion tissues of the IMQ + PCPA group demonstrated a significantly thickened epithelial layer with marked parakeratosis, increased spiny cells, inflammatory cell infiltration, as well as blood vessel hyperplasia and dilation when compared with the control group. Furthermore, we compared the pathological manifestations of lesion tissues between the model and the treatment groups and observed that all treatment groups exhibited varying degrees of improvements in histopathological features compared to the control, consistent with the features detected in skin pathology macroscopically. Notably, the XYAS group demonstrated the most pronounced histopathological improvement, evidenced by two to three epidermal cell layers displaying a notable reduction in parakeratosis and a minimal infiltration of inflammatory cells, along with the most substantial decrease in epidermal thickness. (Figure 1D).

### 3.3 XYAS, LXJD, and QXAS downregulated serum pro-inflammatory cytokines in mice with psoriasis complicated by sleep disturbances

**FIGURE 2:**
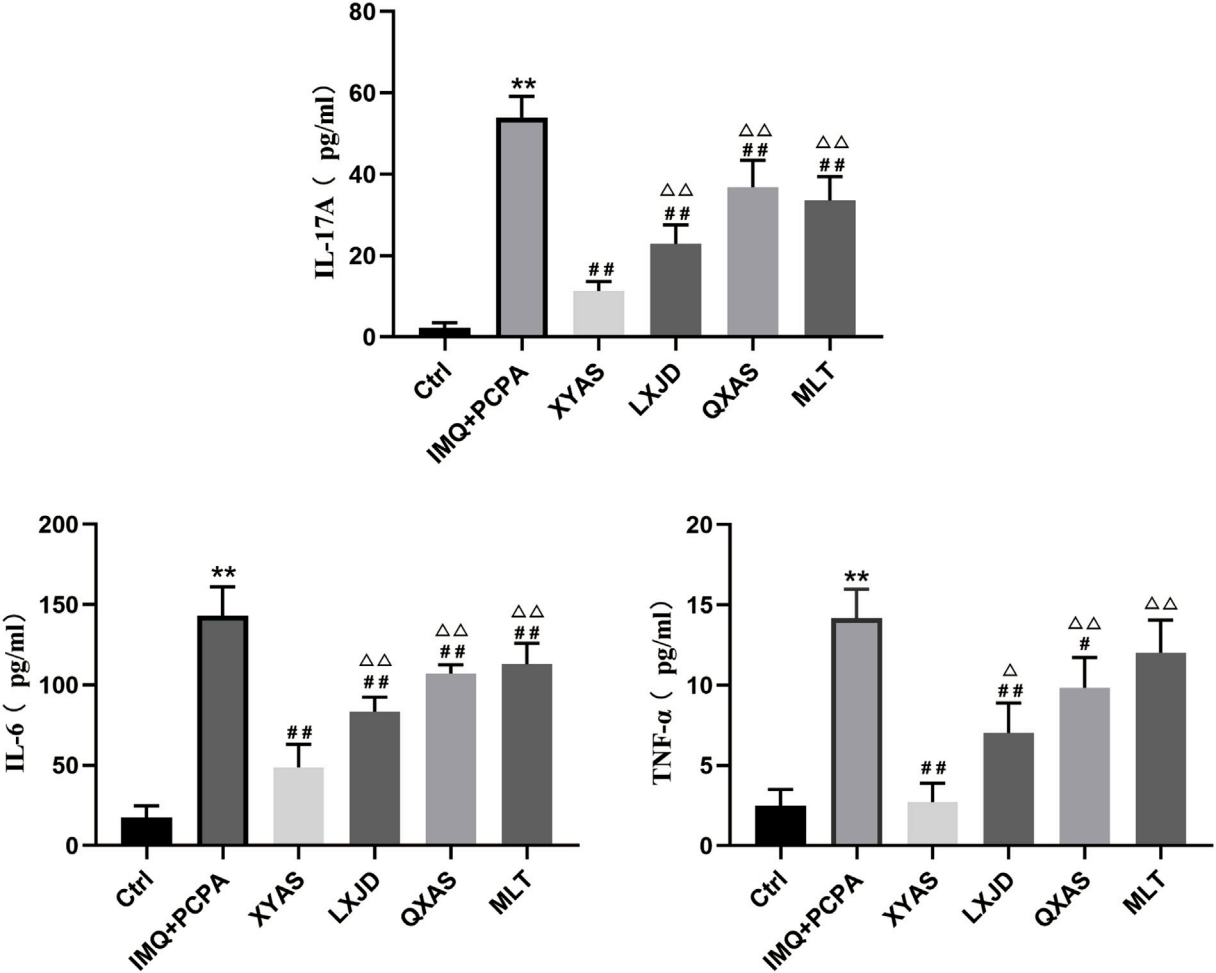
XYAS, LXJD, QXAS, and MLT treatments downregulated serum levels of IL-17A, IL-6, and TNF-α in mice The serum levels of pro-inflammatory cytokines in each group were assessed by ELISA, with the IL-17A shown at the top side, IL-6 shown at the lower left side, TNF-α shown at the lower right side of this figure. ^**^ *P*< 0.01, comparison with the control group; ^#^ *P*< 0.05 or ^##^ *P*< 0.01, comparison with the IMQ + PCPA group; ^△^ *P*< 0.05 or ^△△^ *P*< 0.01, comparisons with the XYAS group.

To further explore the anti-inflammatory effects and mechanisms of XYAS on psoriasis, we examined the expression levels of IL-6, IL-17A, and TNF-α in each group through ELISA at day 14. As shown in Table 2 and Figure 2, the control group exhibited the lowest levels of IL-6, IL-17A, and TNF-α, while the model group displayed the highest levels.Statistically significant differences were observed between these two groups. Compared to the model group, levels of IL-6 and TNF-α gradually decreased in the MLT, QXAS, LXJD, and XYAS groups (Figure 2, lower left side and lower right side), and IL-17A levels gradually decreased in the QXAS, MLT, LXJD, and XYAS groups (Figure 2, top side). Notably, the XYAS group demonstrated the most significant reduction in the levels of all examined cytokines when compared to the model and other treatment groups. The LXJD group exhibited lower IL-6 and IL-17A levels than the AS group (Figure 2, top side and lower left side); however, these two groups showed almost equivalent reductions in TNF-α levels (Figure, lower right side). The MLT group also exhibited significant reductions in IL-6 and IL-17A levels, similar to those observed in the QXAS group (Figure 2, top side and lower left side). However, there was no statistical difference in TNF-α levels between the MLT and model groups (Figure 2, lower right side). These findings indicate that MLT, XYAS, LXJD, and QXAS could inhibit psoriasis-like inflammation to varying degrees by down-regulating the expression levels of IL-6, IL-17, and TNF-α.

**TABLE 2:** The comparisons of serum levels of IL-17A, IL-6, and TNF-α among group**s** (mean ± standard deviation).

Groups	IL-17A (pg/mL)	IL-6 (pg/mL)	TNF-α (pg/mL)
Control	2.18 ± 1.29	17.54 ± 7.27	2.48 ± 1.03
IMQ + PCPA	54.00 ± 5.20	143.10 ± 17.97	14.18 ± 1.80
XYAS	11.28 ± 2.32	48.79 ± 14.28	2.72 ± 1.17
LXJD	22.93 ± 4.65	83.35 ± 9.14	7.03 ± 1.87
QXAS	36.83 ± 6.63	107.00 ± 5.54	9.84 ± 1.89
MLT	31.55 ± 5.92	112.90 ± 13.90	12.00 ± 2.06
*F*	93.50	89.16	46.21
*P*	<0.0001	<0.0001	<0.0001

### 3.4 XYAS, LXJD, and QXAS upregulated MLT and RORα expression levels in psoriatic lesions of mouse models

**FIGURE 3:**
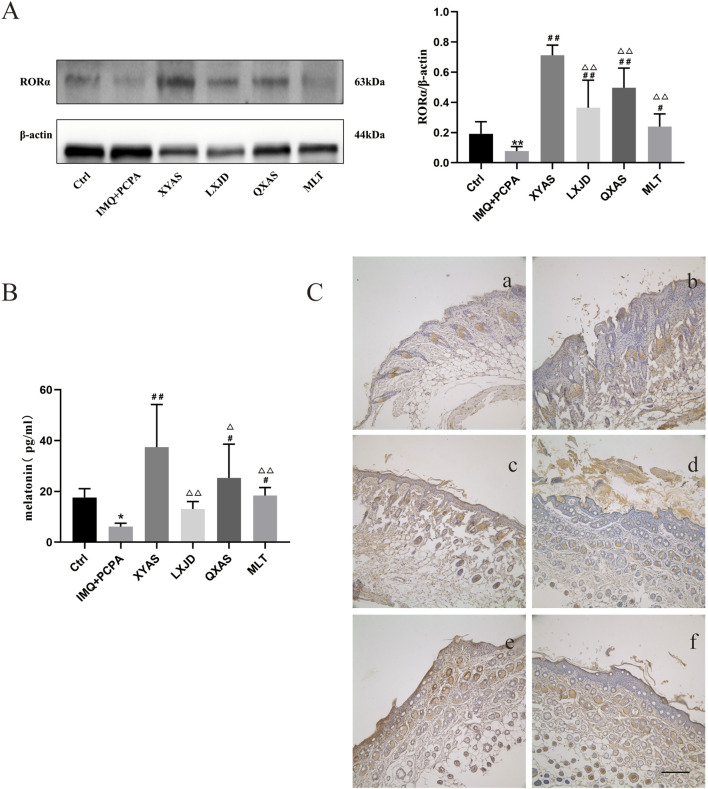
XYAS, LXJD, QXAS, and MLT upregulated the MLT-RORα expression levels in lesion tissues of each group **(A)** Expression levels of RORα in skin tissues assessed by Western blot assay. **(B)** Expression levels of MLT in skin tissues detected by ELISA. **(C)** Immunohistochemical staining for RORα protein localization: Immunohistochemical staining for RORα in back-skin tissues at a magnification of ×100. Brown-yellow granular cells indicate RORα-positive cells. In 3C, a-f represent groups Ctrl (control), IMQ + PCPA, XYAS, LXJD, QXAS, and MLT in turn. **(A, B)** show comparative analyses of RORα and MLT levels across all experimental groups. ^**^ *p* < 0.01, comparison with the control group; ^#^ *p* < 0.05 or ^##^ *p* < 0.01, comparison with the IMQ + PCPA group; ^△^ *p* < 0.05 or ^△△^ *p* < 0.01, comparison with the XYAS group.

We explored the localization and/or levels of MLT and RORα in lesion tissues of each group using ELISA, Western blotting, and immunohistochemical assays at day 14. Notably, the MLT levels were significantly reduced in the IMQ + PCPA group as compared to the control. Meanwhile, the MLT levels gradually increased in the LXJD, MLT, QXAS, and XYAS groups compared to the model, and the differences between the model and the QXAS, MLT, or XYAS groups were statistically significant (Table 3; Figure 3A). Moreover, comparisons of MLT levels between the XYAS and LXJD, QXAS, and MLT groups revealed statistically significant differences. Additionally, the Western blot analysis revealed a significant decrease in RORα level in the model group compared to the control group. Taking model as the comparative reference, a statistically significant and gradual upregulation of RORα levels was observed in the MLT, LXJD, QXAS, and XYAS groups. Additionally, the RORα levels in groups LXJD, MLT, and QXAS differed significantly from those in the XYAS group (Figure 3B). Immunohistochemical analysis demonstrated that RORα, represented by brown-yellow particles, was only expressed in small amounts in hair follicle cells of the model lesions, significantly lower than that in the control group. However, compared with the model, the expression of RORα significantly increased in follicle cells and the stratum corneum of the XYAS and QXAS groups. Moreover, RORα expression increased in hair follicles of lesion tissues in the LXJD and MLT groups compared to the model group but was lower than that in the XYAS group (Figure 3C).

**TABLE 3:** The comparison of melatonin levels in skin tissues of each group of mice (mean ± standard deviation).

Groups	Melatonin (pg/mL)	*F*	*P*
Control	17.61 ± 3.45	3.332	0.018
IMQ + PCPA	6.19 ± 1.29		
XYAS	37.44 ± 16.77		
LXJD	13.14 ± 2.87		
QXAS	25.33 ± 13.31		
MLT	18.37 ± 3.17		

### 3.5 XYAS, LXJD, and QXAS regulated the release of mnSOD and mitochondrial Cyt-C to suppress OS in mice treated with IMQ and PCPA

**FIGURE 4:**
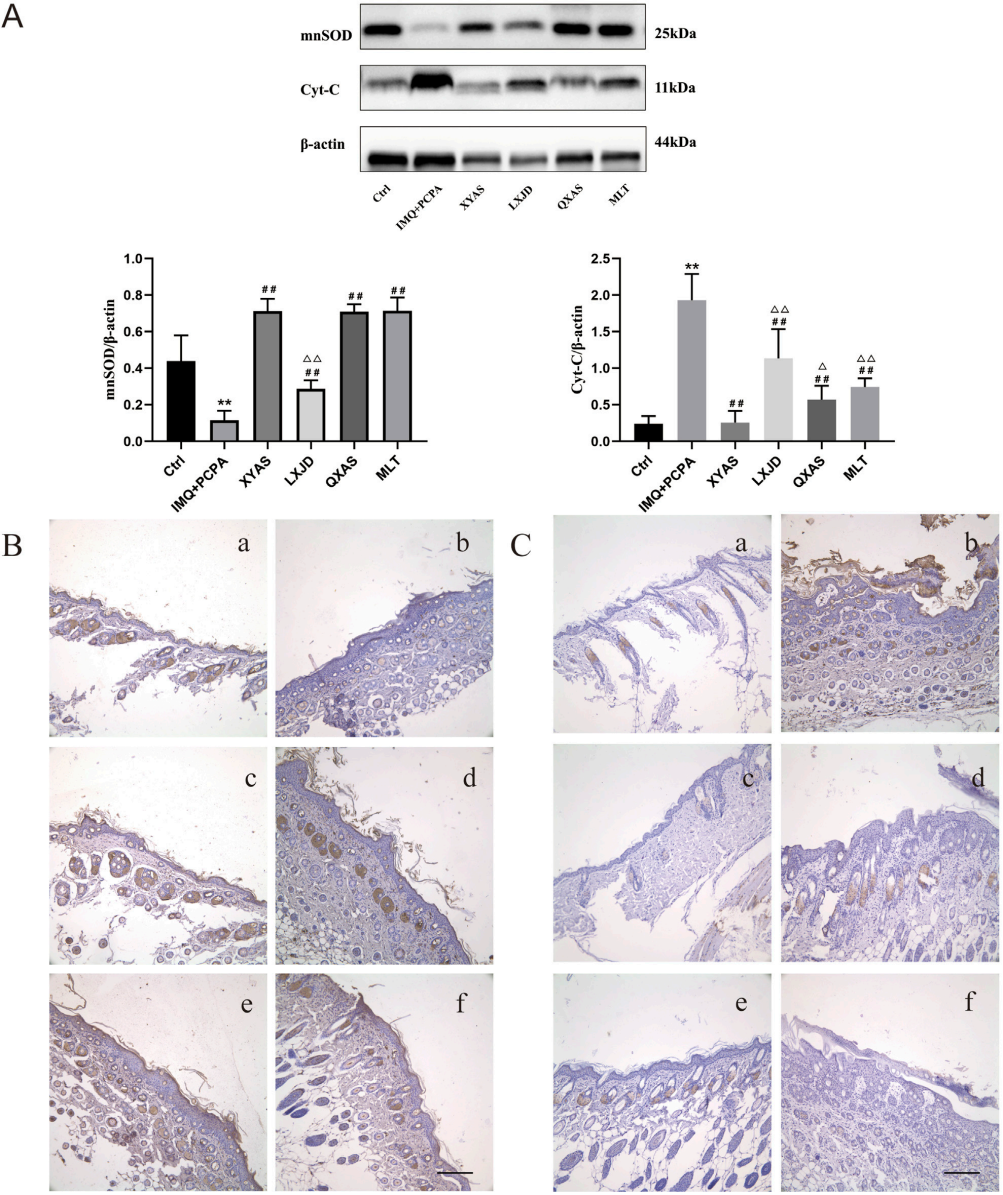
XYAS, LXJD, QXAS, and MLT improved OS and mitochondrial function in lesion tissues of each group of mice **(A)** The mnSOD and Cyt-C expression levels detected by Western blotting: ^**^ *p* < 0.01, comparison with the control group; ^#^ *p* < 0.05 or ^##^ *p* < 0.01, comparison with the IMQ + PCPA group; ^△^ *p* < 0.05 or ^△△^ *p* < 0.01, comparison with the XYAS group. **(B, C)** Immunohistochemical staining for mnSOD and Cyt-C localizations (magnification×100): 4B presents the immunohistochemical staining results for mnSOD, while 4C exhibits the results for Cyt-C. Panels a–f represent groups Control, IMQ + PCPA, XYAS, LXJD, QXAS, and MLT sequentially. Brown-yellow granular cells indicate mnSOD-positive or Cyt-C-positive cells.

Given the crucial role OS plays in the progression of psoriasis by causing persistent skin inflammation and the vicious loop between OS and inflammation (Wu et al., 2020), (Duryee et al., 2021), we examined and compared the levels and localization of mitochondrial Cyt-C and mnSOD in lesion tissues from different groups using Western blotting and immunohistochemical tests. As shown in Western blotting results (Figure 4A), a significant increase in Cyt-C and a decrease in mnSOD expression levels were observed in the model group (IMQ + PCPA) compared to the healthy control. Furthermore, when compared to the model, mnSOD levels in all treatment groups remarkably elevated, while Cyt-C levels gradually and significantly decreased in the LXJD, MLT, QXAS, and XYAS groups. Among these, the XYAS, QXAS, and MLT groups exhibited nearly equivalent levels of mnSOD, which were statistically higher than mnSOD levels in the LXJD group. Moreover, we verified the Western blotting results through immunohistochemical assay. As shown in Figures 4B, C, mnSOD brown-yellow particles were scarcely expressed, while Cyt-C brown-yellow particles were abundantly expressed in hair follicle cells and stratum corneum of the model group compared to the control. Furthermore, compared to the model, mnSOD brown-yellow particles were highly expressed in the hair follicle cells and stratum corneum of mice in the XYAS, QXAS, and MLT groups.And to some extent, the mnSOD brown-yellow particles were observed in hair follicle cells of the mice in LXJD group (Figure 4B). There was almost no significant expression of Cyt-C brown-yellow particles in the hair follicle cells and stratum corneum of the mice in groups XYAS and MLT and only a small amount of Cyt-C brown-yellow particles expressed in hair follicle cells of the mice in the LXJD and QXAS groups.

## 4 Discussion

Psoriasis is frequently accompanied by SDs, leading to increasing immune dysregulation and perpetuated inflammation, thereby fostering a vicious cycle between sleeplessness and psoriasis (Tas et al., 2020; Chang and Chiang, 2018). Several studies have indicated that patients/mice with immune-mediated inflammatory dermatological diseases accompanied by SDs exhibit lower MLT and higher pro-inflammatory cytokines levels compared to those without SDs (Hirotsu et al., 2012; Chang et al., 2014). Additionally, genes associated with mitochondrial biogenesis play a role in balancing ROS production and antioxidant activity, and genes associated with RORα activation are involved in circadian rhythm regulation (Wang et al., 2024). These genes shared between psoriasis and SDs may potentially serve as targets for disease treatment (Kojetin and Burris, 2014; Bin Heyat et al., 2022). Nevertheless, the intricate interaction between MLT, RORα, pro-inflammatory cytokines, antioxidant enzymes, and markers of mitochondrial damage in the pathogenesis of concurrent psoriasis and SDs, as well as whether XYAS can interrupt this vicious cycle by regulating these indicators, remains poorly understood. Our study focused on investigating this interaction in a mouse model of psoriasis combined with SDs to elucidate the pathogenesis of the disease and how XYAS intervenes to reverse psoriasis development. Western blotting and ELISA revealed that the MLT and RORα levels were significantly lower, whereas the pro-inflammatory cytokines and Cyt-C levels were significantly higher in mouse model compared to healthy control. After XYAS treatment, the MLT and RORα levels were significantly elevated, along with an improvement in OS and inflammation. To our knowledge, this is the first research to investigate how TCM impacts the interaction between MLT, RORα, OS, and inflammation in the pathogenesis of psoriasis combined with SDs. The findings suggest that XYAS may disrupt the vicious cycle between psoriasis and SDs by regulating the melatonin-RORα axis, protecting against mitochondrial damage and OS, and inhibiting pro-inflammatory cytokines secretion.

Given the bidirectional relationship between psoriasis and SDs, t is essential to address both SDs and psoriasis in treatment strategies. However, safety concerns regarding current therapies for these diseases present challenges in clinical application. Specifically, some topical treatments for psoriasis may cause skin irritation and burning sensations on lesions, leading to medication non-compliance among patients. Additionally, adverse effects following the administration of oral medications are even more severe than those due to topical treatments (Fouéré et al., 2005). While biologics are highly effective in treating psoriasis, limitations such as inadequate therapeutic responses in some patients (Puig, 2013), decreased efficacy due to neutralizing antibodies generation, the development of neutralizing antibodies (Lee and Kim, 2023), and the high cost of large-scale application remain (Porter et al., 2022). Additionally, benzodiazepine and non-benzodiazepine sedatives are commonly prescribed for insomnia owing to their rapid onset of action (Yang and Deeks, 2012; Lugoboni et al., 2014). However, long-term usage of these medications can trigger drug tolerance and dependence, rebound insomnia after drug withdrawal, and serious inhibition of the central nervous system (Bragg et al., 2019). Furthermore, the role of RORα in immune-mediated dermatological diseases and its involvement in the pathogenesis of psoriasis with SDs necessitates further exploration. Combining the pharmacologic advantages of TCM with its multiple targets, safety profile, affordability (Wang et al., 2022), and remarkable efficacy of XYAS against the co-occurrence of psoriasis and SDs in our clinical study (unpublished data), we established a mouse model of the disease to elucidate the pharmacological mechanisms of XYAS in suppressing inflammation and OS by regulating the aforementioned indicators.

Immune disorders mediated by OS and pro-inflammatory cytokines contribute to the pathogenesis of SDs, which can further exacerbate psoriasis (Halioua et al., 2022; Mullington et al., 2010). Research has shown that sleep deprivation increases pro-inflammatory cytokines such as IL-6 and TNF-α in psoriasis (Hirotsu et al., 2012). Notably, IL-6 and TNF-α levels are elevated in both psoriasis and SDs and have been proven to induce keratinocyte hyper-proliferation in psoriasis (Yang and Deeks, 2012); (Fiedorczuk et al., 2022; Zhang S. et al., 2022). Additionally, in conjunction with IL-6 and TNF-α, IL-23 can stimulate Th17 cells to produce IL-17A, further promoting epidermal hyperplasia and increasing the levels of pro-inflammatory cytokines released from keratinocytes (Medovic et al., 2022). Furthermore, decreased MLT, circadian rhythm disruption, and OS are involved in the pathogenesis of both psoriasis and SDs, with OS being a core pathogenic factor in psoriasis due to the imbalance between mitochondrial damage-induced ROS overproduction and inadequate antioxidant scavenging (Zhang Z. et al., 2022; Song, 2019; Zhang Z. et al., 2023). Subsequently, the damage can result in Cyt-C leakage from mitochondria, a marker of OS and mitochondrial damage (Sidramagowda Patil et al., 2020; Yeh et al., 2022), leading to cell apoptosis, antioxidant system impairment, and mitochondrial dysfunction, which can be represented as increased levels of Cyt-C and decreased levels of SOD and mitochondrial DNA (Wang et al., 2021; Gwozdzinski et al., 2021; Protasoni and Zeviani, 2021). Our *in vivo* animal experiments corroborated these findings, demonstrating significant increases in serum IL-6, IL-17A, TNF-α, and skin Cyt-C levels, along with remarkable reductions in MLT, RORα, and mnSOD expressions in psoriatic lesions compared to healthy controls. Moreover, the results presented in Figures 1B, 3A, B, and 4A indicate that the skin tissue of mice with higher epidermal thickness scores exhibited elevated levels of Cyt-C alongside reduced levels of MLT, RORα, and mnSOD in comparison to those with lower thickness scores. However, Park et al., reported an alleviation of IMQ-induced psoriasis-like symptoms in RORα-deficient mice (Chen et al., 2003). Despite this, García et al., demonstrated that MLT can upregulate RORα levels to boost antioxidant capability and exert an inhibitory effect on NF-κB-mediated inflammation (García et al., 2015), consistent with the low expression levels of MLT and RORα we detected in the lesions of mice with psoriasis + SDs.

Targeted therapies against pro-inflammatory cytokines and OS for psoriasis are rapidly developing. Several studies have demonstrated the efficacy of inhibitors of IL-6, IL-17, and TNF-α in the management of chronic plaque psoriasis and psoriatic arthritis (Hughes and Chinoy, 2013; Bakshi et al., 2020; Tsai and Tsai, 2017). Our findings align with the known functions of IL-6, IL-17, and TNF-α inhibitors, indicating that XYAS, LXJD, and QXAS could significantly reduce IL-6, IL-17, and TNF-α levels and exert an inhibitory effect on epidermal thickening in mice with psoriasis and SDs. MLT, a hormone synthesized in the pineal gland and skin, exerts anti-inflammatory and antioxidant effects on chronic inflammatory dermatological diseases by scavenging ROS, increasing the activity of antioxidant enzymes, and decreasing levels of pro-inflammatory cytokines such as IL-6 and TNF-α (Jaworek et al., 2021; Reiter et al., 2000; Ahmad et al., 2022). Moreover, Scuderi et al., and Kobylińska et al., have demonstrated that MLT can inhibit psoriasis-like skin inflammation and reduce Cyt-C release by increasing ROS removal (Scuderi et al., 2022; Kobylińska et al., 2017). MLT also activates RORα to suppress NF-κB-mediated inflammation and alleviate OS via mnSOD upregulation (Yeh et al., 2022); (Xu et al., 2019). These findings are consistent with our observations in mice, where MLT treatment significantly increased RORα and mnSOD levels while reducing Cyt-C expression levels in skin lesions, along with a decrease in serum IL-6, IL-17, and TNF-α levels. Additionally, we discovered that XYAS has the potential to increase skin MLT-RORα expressions to protect mitochondrial integrity and improve antioxidant capacity, thereby inhibiting psoriatic inflammation, as demonstrated by increased levels of mnSOD and decreased levels of Cyt-C in skin lesions, along with a remarkable increase in serum IL-6, IL-17, and TNF-α levels.

In our study, we found that LXJD and QXAS in XYAS targeted different therapeutic aspects for psoriasis combined with SDs. The LXJD group exhibited significant reductions in serum IL-17A, IL-6, and TNF-α levels, along with a remarkable increase in mnSOD expression and a decrease in Cyt-C expression in lesions compared to those in the QXAS group. Additionally, LXJD treatment significantly improved scaling compared to QXAS treatment. Compared to the LXJD group, the QXAS group demonstrated significant improvements in the expression levels of MLT, RORα, and mnSOD, along with a significant decrease in Cyt-C levels and a significant reduction in epidermal thickness in lesion tissues. Notably, the XYAS group exhibited the best therapeutic effects on reducing disease severity, enhancing antioxidant and anti-inflammatory capabilities, and improving MLT and RORα expression in the skin. These findings suggest that LXJD acts on the disease mainly by inhibiting the generation of, while QXAS plays a protective role against OS and mitochondrial damage by improving the MLT-RORα axis. Importantly, XYAS, consisting of LXJD and QXAS, can take advantage of synergies between LXJD and QXAS in terms of inhibition of keratinocyte proliferation, pro-inflammatory cytokines secretion, and OS in treating psoriasis complicated by SDs.

The present study confirmed the therapeutic effects and uncovered the pharmacological mechanism of XYAS in disrupting the vicious cycle between psoriasis and SDs. However, it's essential to acknowledge two limitations in our study: First, the TCM formulae were not quantified by ultrahigh performance liquid chromatography-tandem mass spectrometry (UPLC-MS/MS) method; Second, other pharmacological mechanisms of XYAS may be involved and thus further exploration is required. As a result, it is necessary to identify active ingredients in XYAS by using UPLC-MS/MS method and expand the understanding of XYAS’ mechanism through network pharmacology and molecular research in our fellow-up study.

## 5 Conclusion

Overall, our findings suggest that XYAS can alleviate psoriasis like-inflammation and abnormal skin morphology in mice with psoriasis complicated by SDs, the pharmacological mechanism of which is possibly related to skin MLT-RORα upregulation, antioxidant activities enhancements, and a suppression of mitochondrial damage. As components of XYAS, LXJD was proven to possess significant anti-inflammatory and antioxidant abilities, while QXAS could improve MLT-RORα secretion and antioxidant activity in skin. Taken together, LXJD and QXAS exert a synergistic role in enhancing skin MLT-RORα expressions, improving antioxidant activity, strengthening mitochondrial protection, and inhibiting the release of pro-inflammatory cytokines, including IL-17A, IL-6, and TNF-α. Consequently, our findings reflect the characteristics of XYAS’ compositions based on mind-body unity and verified its unique advantages in treating psoriasis with overall regulation and multi-targets.

## Data Availability

The raw data supporting the conclusions of this article will be made available by the authors, without undue reservation.
